# Self-Powered Nitrogen Dioxide Sensor Based on Pd-Decorated ZnO/MoSe_2_ Nanocomposite Driven by Triboelectric Nanogenerator

**DOI:** 10.3390/nano12234274

**Published:** 2022-12-01

**Authors:** Weiwei Wang, Dongyue Wang, Xixi Zhang, Chunqing Yang, Dongzhi Zhang

**Affiliations:** College of Control Science and Engineering, China University of Petroleum (East China), Qingdao 266580, China

**Keywords:** TENG, self-powered, NO_2_ gas sensor, ternary nanocomposite

## Abstract

This paper introduces a high-performance self-powered nitrogen dioxide gas sensor based on Pd-modified ZnO/MoSe_2_ nanocomposites. Poly(vinyl alcohol) (PVA) nanofibers were prepared by high-voltage electrospinning and tribological nanogenerators (TENGs) were designed. The output voltage of TENG and the performance of the generator at different frequencies were measured. The absolute value of the maximum positive and negative voltage exceeds 200 V. Then, the output voltage of a single ZnO thin-film sensor, Pd@ZnO thin-film sensor and Pd@ZnO/MoSe_2_ thin-film sensor was tested by using the energy generated by TENG at 5 Hz, when the thin-film sensor was exposed to 1–50 ppm NO_2_ gas. The experimental results showed that the sensing response of the Pd@ZnO/MoSe_2_ thin-film sensor was higher than that of the single ZnO film sensor and Pd@ZnO thin-film sensor. The TENG-driven response rate of the Pd@ZnO/MoSe_2_ sensor on exposure to 50 ppm NO_2_ gas was 13.8. At the same time, the sensor had good repeatability and selectivity. The synthetic Pd@ZnO/MoSe_2_ ternary nanocomposite was an ideal material for the NO_2_ sensor, with excellent structure and performance.

## 1. Introduction

Nitrogen oxide (NO_2_) is an important component on air pollutants, which has a serious impact on human health. Among them, the exhaust gas from motor vehicles and power plants in industry releases more nitrogen dioxide gas [1]. NO_2_ can stimulate people’s respiratory tract, reduce the resistance of the respiratory tract and induce various inflammation of the respiratory tract. Moreover, it participates in photochemical reactions and forms acid rain, which is harmful to ground organisms and human health [2]. Therefore, the sensitive NO_2_ gas sensor has attracted wide attention [3]. So far, in various sensing technologies, many scholars have used metal oxide semiconductors (such as WO_3_, ZnO, CuO, In_2_O_3_) to prepare gas sensors [4,5,6]. However, most metal oxides have great requirements for temperature conditions when detecting NO_2_, so it is necessary to develop a NO_2_ sensor at room temperature. ZnO has unique properties and has broad application prospects in sensors, thin-film transistors, light-emitting diodes and other fields. It is sensitive to NO_2_ gas at RT and widely used as a sensing material. The catalysis of noble metal can reduce the activation energy of the reaction, greatly improving the selectivity and sensitivity of gas sensors [7]. It is particularly important to develop sensors with high sensitivity, low cost and miniaturization. The interdigital electrode can be used for various miniaturized sensors because of its tiny electrode spacing structure. Traditional analytical detection, including chromatography, spectroscopy, mass spectrometry and other methods, mostly need expensive instruments and a variety of operating steps, leading to many practical problems and difficulties. At the same time, the interdigital electrode is a parallel circuit, which can increase the response to the test signal and reduce the manufacturing cost of the sensor.

In recent years, wind power, hydropower and solar power have brought great convenience to people’s life, but they are more vulnerable to the weather, bulky and not easy to carry [8]. In 2012, Professor Wang also proposed triboelectric nanogenerators (TENGs) [9]. A nanogenerator has a simple structure and low cost and can collect energy from the environment. In recent years, nanogenerators have been widely studied and many achievements have been made in applications [10,11]. The TENG is usually composed of triboelectric pairs and corresponding conductive electrodes. The potential is generated during friction. In order to make the potential equal, the charge (electron or ion/molecule) will move between the two materials [12,13,14]. Common friction materials, such as polytetrafluoroethylene (PTFE), polyimide film (PI), polydimethylsiloxane (PDMS) and so on, have been widely used in TENGs [15]. With the continuous development of science and technology, nanogenerators can be combined with sensors to realize the development of self-powered sensors, which provides new insights for the development of sensors [16,17]. 

In this work, PVA was synthesized on aluminum foil (Al-PVA) by electrospinning and a nanogenerator was prepared by using Al-PVA and PI as friction materials. What is more, using it for a Pd@ZnO/MoSe_2_-based NO_2_ gas sensor provides power at 25 °C. In this paper, the voltages of TENGs at different frequencies were measured. The TENG was used as the power supply. The sensing performance of the NO_2_ sensor was investigated towards NO_2_ gas. Compared with single ZnO and Pd@ZnO, the sensing properties of Pd@ZnO/MoSe_2_ were improved obviously. 

## 2. Experimental

### 2.1. Materials

Reagents used in the experiments mainly included zinc nitrate hexahydrate (Zn(NO_3_)_2_·6H_2_O), sodium dodecyl sulfate (SDS), polyvinyl alcohol (PVA), palladium chloride (PdCl_2_), sodium hydroxide (NaOH), sodium molybdate dihydrate (Na_2_MoO_4_·2H_2_O), selenium powder (Se), sodium borohydride (NaBH_4_), ethanol and hydrazine hydrate, which were obtained from Sinopharm Chemical Reagent Co. Ltd.

### 2.2. Material Synthesis

Electrospinning synthesis of PVA: 3.9 g PVA was added into 30 mL DI water under stirring for 30 min at 25 °C, and 0.1 g SDS powder was added into the above solution. The SDS is beneficial to the formation of nanofibers [18,19]. The 11 wt% of PVA solution was continuously stirred at 90 °C for 6 h. Al substrate was attached to the disc to collect the electrospun fiber film [20]. PVA nanofiber network has certain viscoelasticity and can recover after deformation, so the TENG can maintain good frictional durability.

Synthesis of ZnO nanoparticles (NPs): 2.08 g Zn (NO_3_)_2_·6H_2_O was added to 140 mL deionized (DI) water and stirred for 30 min at 25 °C. Then, 3.2 g NaOH was dissolved into 20 mL DI water to prepare 4 mol/L aqueous solution. Then the two aqueous solutions were mixed under continuous stirring for 1 h and ultrasonic vibration for 15 min. Subsequently, the mixed solution was transferred into an autoclave and heated at 120 °C for 12 h. Finally, the ZnO nanoparticles were obtained by centrifugation and washing with DI water and dried at 60 °C overnight. 

Synthesis of Pd@ZnO NPs: Certain amounts of PdCl_2_, hydrazine hydrate (20 μL) and NaOH (50 mg) were dissolved in 80 mL DI water under continuous stirring for 1 h, and hydrazine hydrate was used to obtain reduced palladium from PdCl_2_. Then, the prepared ZnO was added into the above solution and heated in an autoclave at 180 °C for 10 h. Finally, the Pd@ZnO product was treated by centrifugation and washing with DI water and dried at 60 °C overnight [21].

**Figure 1:**
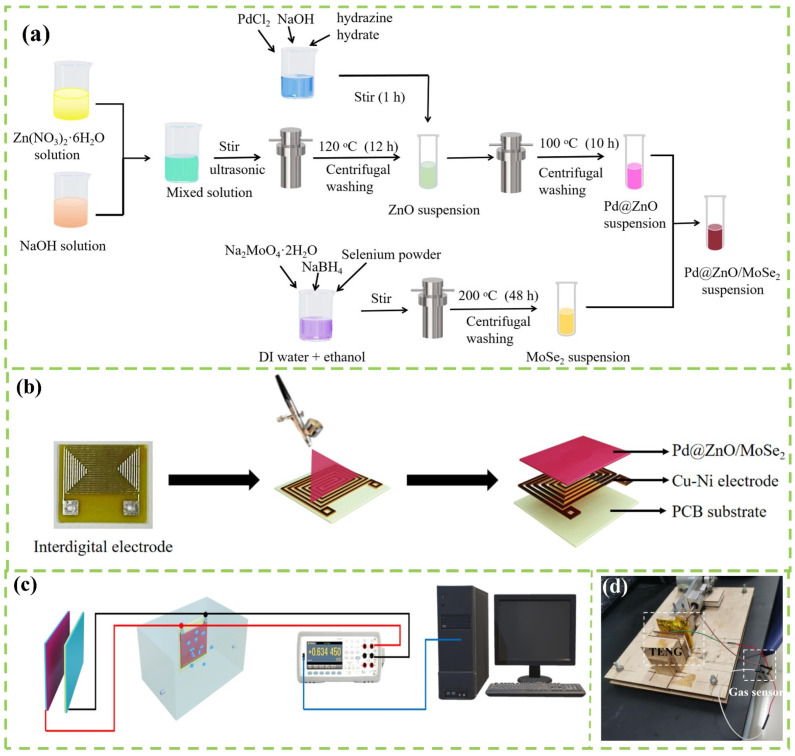
Manufacturing process of (**a**) Pd@ZnO/MoSe_2_ and (**b**) gas sensor. (**c**) A diagram of the test system. (**d**) A picture of the TENG and the sensing device powered with TENG.

Synthesis of Pd@ZnO/MoSe_2_ film sensor: MoSe_2_ nanoflowers were synthesized by solvothermal reaction. Na_2_MoO_4_·2H_2_O (0.6 g) and NaBH_4_ (0.1 g) were dissolved in a mixed solution of 25 mL deionized water and 25 mL ethanol. Then, 10 mL of hydrazine hydrate solution containing 0.493 g selenium powder was poured into the above solution and stirred violently at 25 °C for 2 h. Then, the solution was put into a reactor and heated at 200 °C for 48 h. The black MoSe_2_ powder was finally collected by washing with ethanol and deionized water several times and dried overnight at 60 °C. The precipitate was washed with deionized water and methanol, and the unreacted monomer was removed with acetone many times. To obtain Pd@ZnO/MoSe_2_ solution, 0.2 g MoSe_2_ powder was added to the pre-prepared Pd@ZnO solution under continuous ultrasonic dispersion for 1 h. Finally, the solution was dripped onto the interdigital electrode and dried at 60 °C for 4 h to form a sensing film. The detailed manufacturing process of the thin-film sensor is shown in Figure 1a. The interdigital electrodes (IDEs) were prepared by sputtering, photolithography and development. As shown in Figure 1b, the gas-sensing materials were deposited on the IDEs via spraying. The dimension of IDEs is 8 × 8 mm and the thickness is 300 μm. The NO_2_ gas-sensing characteristics of the sensor were investigated by studying the response, response/recovery time and selectivity of the sensor. A diagram of the test system is shown in Figure 1c. The sensor as a load was plugged into the TENG circuit. The sensor response can be defined as follows: Response = V_g_/V_a_, where V_a_ and V_g_ represent the voltage across the sensor when the sensor is in air and NO_2_ gas, respectively. The response/recovery time at a specific concentration is defined as the time for sensor to achieve 90% of maximum response.

### 2.3. Sensor Assembly

**Figure 2:**
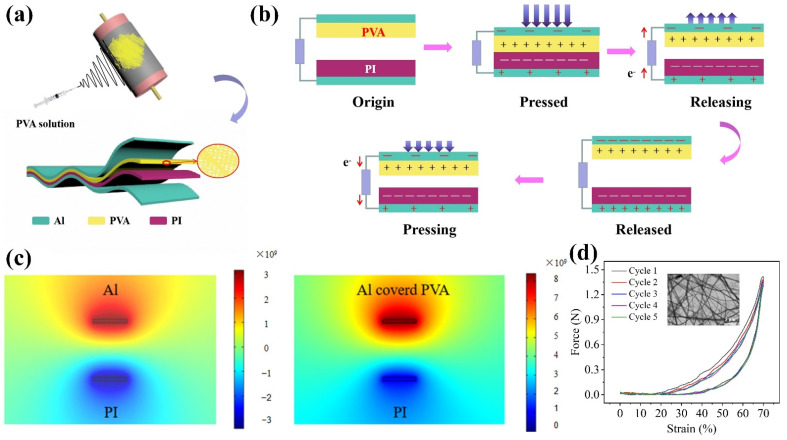
(**a**) Structure of Al-PVA/PI TENG and manufacturing process. (**b**) Schematic diagram of operating principle of generator. (**c**) Effect of TENG friction potential. (**d**) Hysteresis cycle of PVA on TENG surface.

Figure 2a shows the design structure of the Al-PVA/PI triboelectric nanogenerator. The structure of TENG mainly consists of two parts: one is electrode and the other is friction material. Al layer acted as electrode, PVA was covered on Al electrode and polyimide film was regarded as friction layer. The nanogenerator was used to drive the synthesized NO_2_ sensor, in which the sensing characteristics of the thin-film sensor were tested on exposure to NO_2_ gas at 25 °C. In order to test the voltage signal and the gas response signal, the device was connected with the key 34,470 A.

### 2.4. Characterization Instrument

The morphology and microstructure of nanocomposites were studied by using a scanning electron microscope (SEM, HITACHI S-4800). X-ray diffraction (XRD) was measured to clarify phase composition and purity of samples on RIGAKU D/max 2500 PC. X-ray photoelectron spectroscopy (XPS; Thermo Scientific escalator 250Xi) was performed to investigate elemental composition and chemical valence states of Pd@ZnO/MoSe_2_ samples.

## 3. Results and Discussion

### 3.1. Characterizations of TENG

Figure 2b illustrates a schematic diagram of the operating principle of the generator. According to the triboelectric sequence [12], PVA is more likely to lose electrons after contact with PI. In the original state, there is no potential difference between the two electrodes or induced charges. When the two films are separated, a potential difference is formed between the two films and drives electrons from the electrode on the PI to the electrode on the PVA via the external load. When two friction material surfaces contact again, a reverse current is generated. Figure 2c shows the effect of TENG friction potential studied by analysis and simulation using COMSOL software. Figure 2d illustrates the morphology of PVA on the surface of the TENG. The PVA is mainly composed of nanofibers with an average diameter of 550 nm.

**Figure 3:**
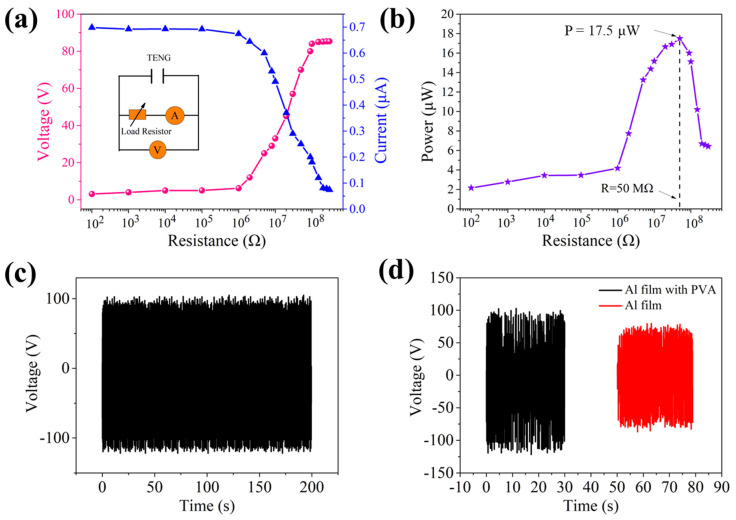
(**a**) Under the TENG, dependence of output voltage and current on external loading resistance. Insert is measurement circuit diagram. (**b**) Plot of output power versus loading resistance. (**c**) Repeatability of TENG at 10 Hz. (**d**) Comparison of output voltage of TENG with PVA and that without PVA at 10 Hz.

Figure 3a,b illustrate the dependence of output voltage, current and output power of the TENG on resistance. The results show that the output voltage increases with the increase in load resistance, while the output current is the opposite. The maximum output power can reach 17.5 μW. Figure 3c shows that the mechanical properties of the TENG are relatively stable at 10 Hz, and the sum of absolute values of positive and negative maximum voltages is greater than 200 V. Figure 3d explains that the output voltage of TENG with PVA is much higher than that without PVA at 10 Hz, which is consistent with the simulation results. Compared with the pure Al film, the introduction of PVA nanofibers greatly improves the output performance of the TENG. PVA tends to lose electrons, which may generate more triboelectric charges. In addition, the nanofiber network structure improves the effective contact area of the material.

### 3.2. Sample Characterization

**Figure 4:**
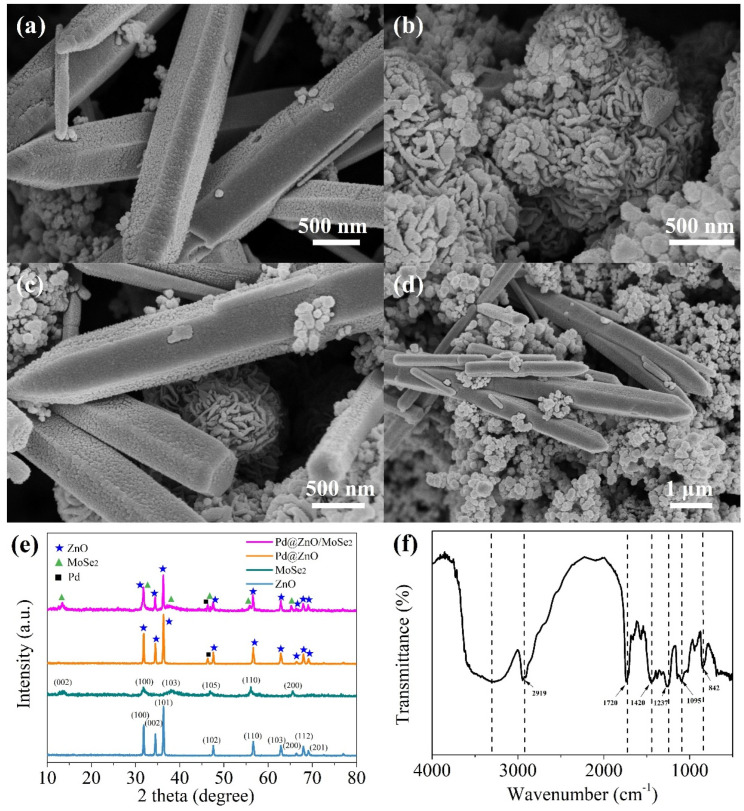
SEM images of (**a**) pure ZnO nanofibers, (**b**) pure MoSe_2_ nanoflowers and (**c**,**d**) Pd@ZnO/MoSe_2_. (**e**) FTIR spectrum of PVA samples. (**f**) XRD of ZnO, MoSe_2_, Pd@ZnO and Pd@ZnO/MoSe_2_ samples.

Figure 4a,b describe the SEM images of pure ZnO and MoSe_2_ samples. Zinc oxide has a rod structure and the MoSe_2_ sample is mainly composed of nanoflowers self-assembled by nanosheets. Figure 4c,d show the SEM images of Pd@ZnO/MoSe_2_ composites. It can be observed that the composites have good contact with each other, which is conducive to obtaining a large number of gas-diffusion pathways.

The FTIR spectrum of PVA nanofibers in a range of 4000–500 cm^−1^ is shown in Figure 4e. The broad peak at 3338 cm^−1^ is due to the stretching vibration of the O–H group of PVA. The peak at 2919 cm^−1^ is attributed to the asymmetric stretching mode of C–H. The peak at 1720 cm^−1^ is attributed to the bending vibration of C=O and the band at 1420 cm^−1^ is attributed to C–H bending. The peaks at 1237, 1095 and 842 cm^−1^ are attributed to C–C–O, C–O and C–C stretching, respectively [22]. Figure 4f illustrates the XRD spectra of ZnO, MoSe_2_, Pd@ZnO and Pd@ZnO/MoSe_2_ samples. For ZnO, the diffraction peaks at 31.8°, 34.3°, 36.3°, 47.6°, 56.6°, 62.9°, 66.3°, 67.9° and 69.1° are obviously observed, respectively, corresponding to (100), (002), (101), (102), (110), (103), (200), (112) and (201) planes (JCPDS Card No. 36-1451) [23]. The diffraction peaks of MoSe_2_ at 13.36°, 31.53°, 37.26°, 46.97°, 55.95° and 65.72° are shown, respectively, corresponding to (002), (100), (103), (105), (110) and (200) planes (JCPDS Card No. 29-0914) [20]. The diffraction peak of Pd@ZnO at 46.53° proves the existence of Pd [24]. The XRD pattern of Pd@ZnO/MoSe_2_ nanocomposites contains the diffraction peaks of the three components, indicating the existence of Pd@ZnO/MoSe_2_ nanocomposites.

**Figure 5:**
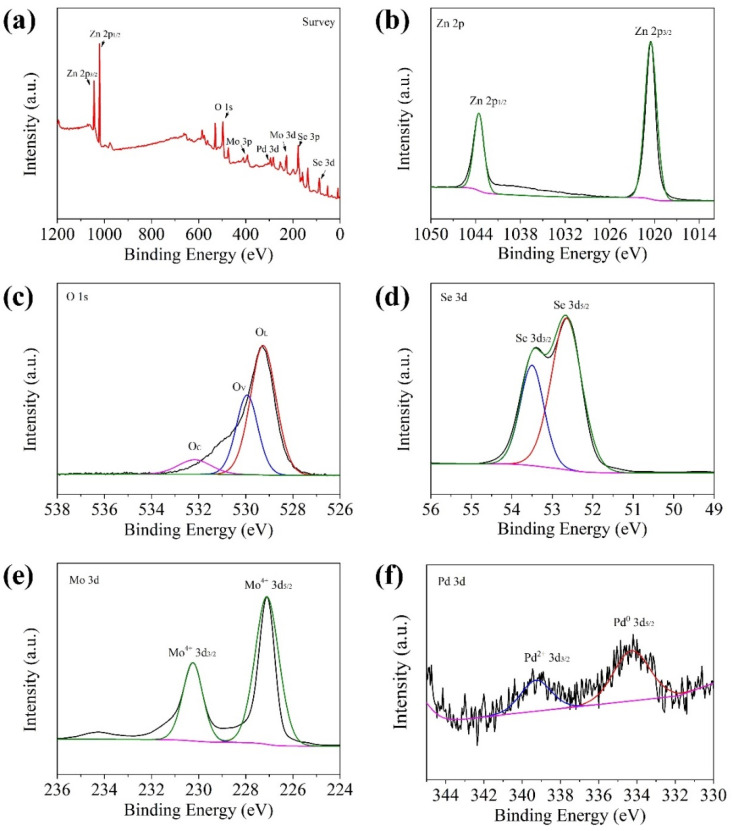
XPS spectra of Pd@ZnO/MoSe_2_ sample: (**a**) full spectrum, (**b**) Zn 2p spectrum, (**c**) O 1s spectrum, (**d**) Se 3d spectrum, (**e**) Mo 3d spectrum and (**f**) Pd 3d spectrum.

The surface composition of the Pd@ZnO/MoSe_2_ sample was further analyzed by XPS technology, as shown in Figure 5. The full spectrum of the Pd@ZnO/MoSe_2_ sample in Figure 5a demonstrates that the sample is composed of the five elements of Pd, Zn, Mo, Se and O. Figure 5b shows the spectrum of Zn 2p. Two characteristic peaks at 1021.5 and 1044.3 eV, respectively, show the spin-orbit dipole splitting of Zn 2p_1/2_ and Zn 2p_3/2_. Figure 5c shows the O1s spectrum. The characteristic peaks at 529.3 and 530.27 eV can be attributed to lattice oxygen (O_L_) and ZnO-OH (O defect) in the Zn-O bond (O_V_), and the characteristic peak at 531.8 eV can be attributed to the chemical-absorbed oxygen (O_C_) [25]. The Se 3d spectrum in Figure 5d shows that two characteristic peaks at about 52.6 and 53.46 eV can be assigned to Se 3d_5/2_ and Se 3d_3/2_, respectively. The Mo 3d spectrum in Figure 5e shows that two characteristic peaks at energy band energies of 227.3 and 230.25 eV are attributed to Mo 3d_5/2_ and Mo 3d_3/2_ of the Mo^4+^ state in MoSe_2_, respectively. Figure 5f shows the core energy level spectrum of Pd 3d. The peaks at 335.33 and 341.57 eV are assigned to Pd^0^ and Pd^2+^, respectively [23,26].

### 3.3. Sensing Properties

**Figure 6:**
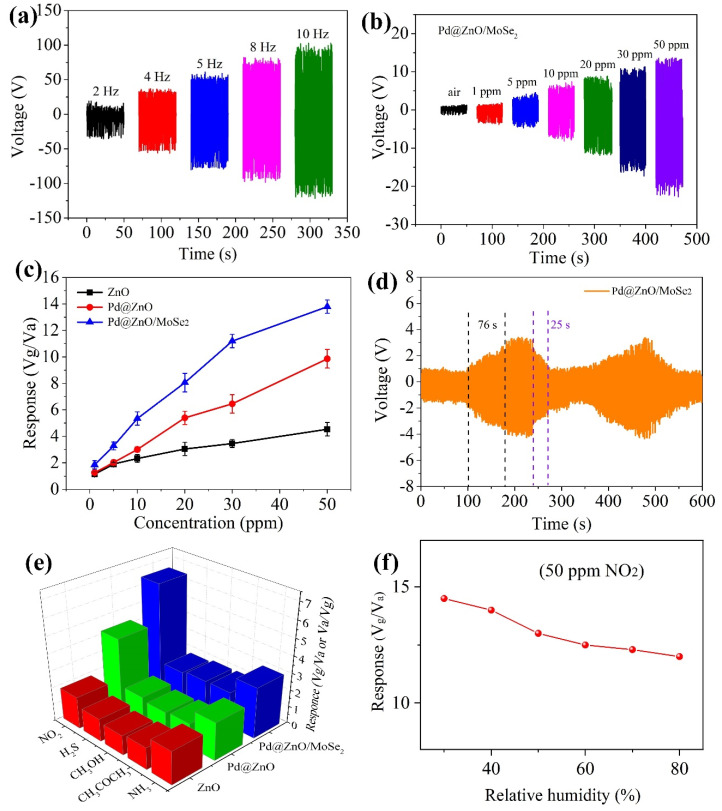
(**a**) Voltage test diagram of TENG at different frequencies at 25 °C. (**b**) Voltage values of Pd@ZnO/MoSe_2_ exposed to different concentrations of NO_2_ at 5 Hz at 25 °C. (**c**) Response fitting curves of ZnO, Pd@ZnO and Pd@ZnO/MoSe_2_ film sensors toward different concentrations of NO_2_ gas. (**d**) Mechanical reliability test of Pd@ZnO/MoSe_2_ composite exposed to 1 ppm NO_2_ at 5 Hz. (**e**) Response of Pd@ZnO/MoSe_2_ exposed to 10 ppm nitrogen dioxide, hydrogen sulfide, methanol, acetone and ammonia. (**f**) The sensor response in different humidity levels.

Figure 6a illustrates the output voltage of the TENG at room temperature (RT) in air at different frequencies (2, 4, 5, 8 and 10 Hz). It can be seen from the figure that the output voltage of the TENG increases with the increase in frequency. When the frequency reaches 10 Hz, the output voltage reaches the maximum. The sum of absolute values of positive and negative voltages of TENG is about 200 V at 10 Hz. On this basis, the NO_2_ sensing performances of the ZnO, Pd@ZnO and Pd@ZnO/MoSe_2_ film sensors were studied by using the TENG as a power supply. Figure 6b shows the changes in output voltages of the three sensors when exposed to NO_2_ gas with a concentration of 1–50 ppm at a frequency of 5 Hz. With the increase in the NO_2_ concentration, the output voltage values of all the three sensors increase significantly [27,28]. When the sensors are in the air, peak-to-peak voltages of the ZnO, Pd@ZnO and Pd@ZnO/MoSe_2_ sensors are 7.5 V (±0.6 V), 2.2 V (±0.3 V) and 2.4 V (±0.2 V), respectively. When the sensors are in the 50 ppm NO_2_, peak-to-peak voltages of the ZnO, Pd@ZnO and Pd@ZnO/MoSe_2_ sensors are 33.95 V (±2.8 V), 21.69 V (±2.5 V) and 33.14 V (±2.7 V), respectively. Based on the definition, the voltage responses of the three self-powered sensors at different concentrations of NO_2_ gas are calculated, as shown in Figure 6c. The response of the Pd@ZnO/MoSe_2_ sensor based on the TENG towards 50 ppm NO_2_ gas reaches 13.8. Obviously, the response of Pd@ZnO/MoSe_2_ is higher than that of ZnO and Pd@ZnO. In addition, Figure 6d shows that the response and recovery times of the Pd@ZnO/MoSe_2_ sensor exposed to 1 ppm NO_2_ gas are 76 and 25 s, respectively. The recovery time of the sensor is shorter than the response time, which may be because sensitive materials require less energy during desorption. At the same time, it also shows that the sensor has good recovery performance and mechanical stability. Figure 6e shows the responses of the Pd@ZnO/MoSe_2_ thin-film sensor exposed to 10 ppm hydrogen sulfide, methanol, nitrogen dioxide, acetone and ammonia. The thin-film sensor has the highest response to NO_2_ gas, indicating good selectivity to NO_2_. In addition, Table 1 shows the comparison of the sensing performance of the NO_2_ sensors prepared in this work and previously reported [29,30,31,32]. The results show that the Pd@ZnO/MoSe_2_ composite film-based sensor has a high response. The sensor response in different humidity levels is shown in Figure 6f. The sensor response gradually decreases with the increase in humidity level.

### 3.4. Wearable Practical Application

**Figure 7:**
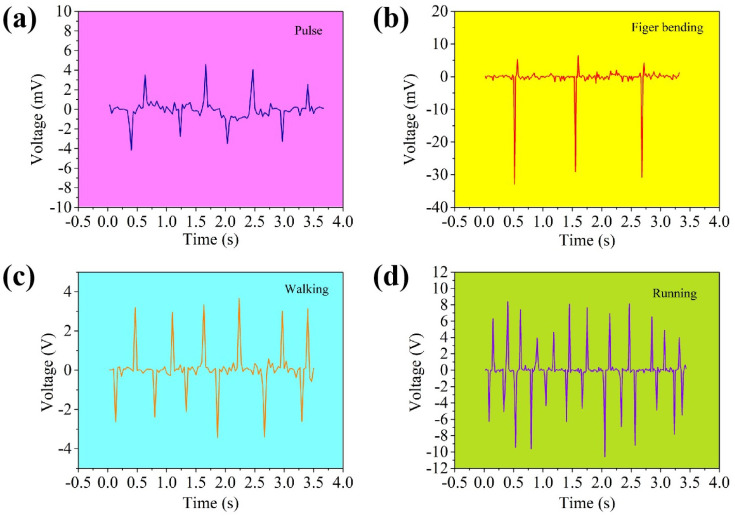
Wearable nanogenerator based on TENG at 25 °C: (**a**) placed on the wrist for weak pulse signals, (**b**) placed on the index knuckle to bend and clench and (**c**,**d**) placed on the sole of the foot to walk and run to generate energy.

It is found that the TENG can generate different energy driven by human motion. The device was placed separately on the different parts of the human body and the movement of the body part can produce a real-time voltage response. As shown in Figure 7a, the device is placed on the wrist to obtain the regular pulse signal of the human body. Figure 7b shows that the device at the joint of the index finger generates an approximate voltage signal when the finger is bent. The device was placed on the insole on the sole of the foot to observe the real-time output voltage of walking slowly (Figure 7c) and running (Figure 7d). Running can generate about 7 V voltage signal, which provides a new idea for the research of self-powered wearable devices.

## 4. NO_2_ Sensing Mechanism

**Figure 8:**
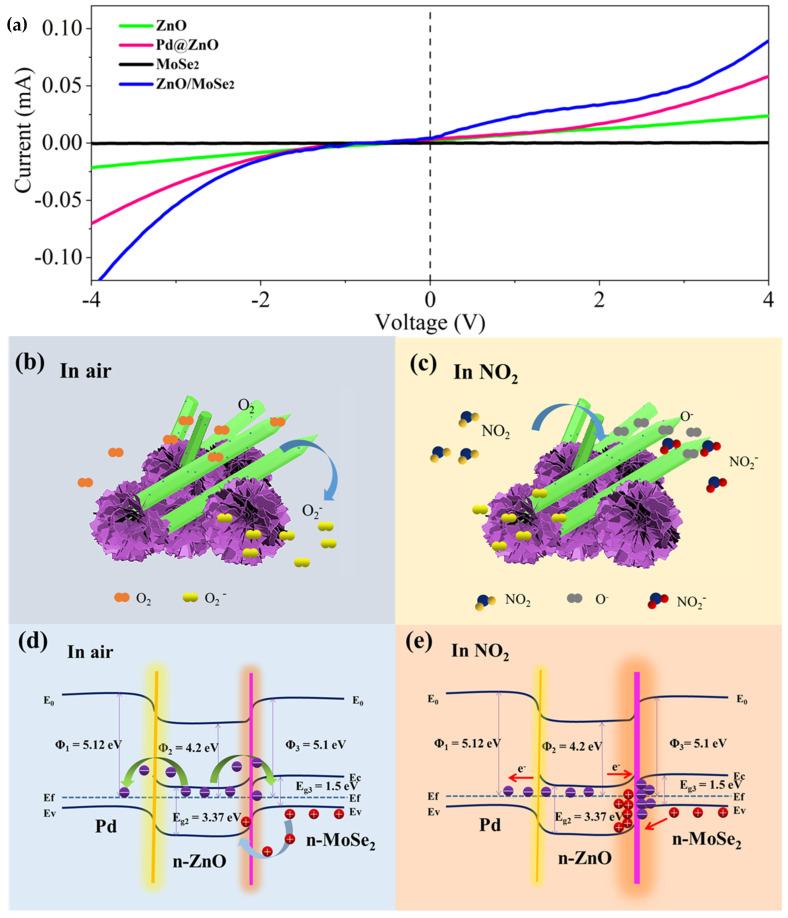
(**a**) I–V curve of ZnO, MoSe_2_, Pd@ZnO and ZnO/MoSe_2_ at 25 °C. (**b**,**c**) Gas-sensing mechanism of Pd@ZnO/MoSe_2_ composites in air and NO_2_. (**d**,**e**) Energy band structure of Pd@ZnO/MoSe_2_ composites in air and NO_2_ (E_f_, Fermi-energy level; E_C_, conduction band; E_V_, valence band; φ, work function).

Figure 8a shows the I–V curves of ZnO, MoSe_2_, Pd@ZnO and ZnO/MoSe_2_ thin films. The I–V characteristic curve of the Pd@ZnO sensor shows obvious nonlinearity between −4 and 4 V, which reveals the good Schottky contact between ZnO nanoparticles and Pd. The slope of the I–V curve also shows that the conductivity of the Pd@ZnO sensing film is much higher than that of ZnO [33]. Pd nanoparticles dispersed on the surfaces of nanocomposites can enhance the availability of active sites for gas adsorption, which is conducive to gas adsorption. At the same time, ZnO/MoSe_2_ thin films have better nonlinearity than single ZnO and MoSe_2_, which indicates that the heterojunction has better rectification performance [34].

From the experimental results, we can find that the response of the Pd@ZnO/MoSe_2_ composite thin-film-based sensor to NO_2_ gas is obviously improved at room temperature. The gas-sensing mechanism of semiconductors can be explained by the chemical adsorption of gas molecules and the electron exchange caused by oxygen adsorption [35]. Figure 8b,c show the Pd@ZnO/MoSe_2_ sensing mechanism in air and NO_2_ gas at room temperature. When the sensor is exposed to the air, oxygen molecules in the air will be adsorbed on the surface of the sensing material [36]. The oxygen molecules capture electrons and produce chemically adsorbed oxygen anions (O_2_^−^). 

When the sensor is exposed to NO_2_ gas, the highly electrophilic NO_2_ molecules extract electrons directly from the sensing layer and react with O_2_^−^, resulting in an increase in sensor resistance. When the sensor is switched to the air, the electrons trapped by NO_2_ molecules are released back to the conductive band of the material, causing the resistance to return to its initial state. The process is as follows [32]:

O_2_ (gas) → O_2_ (ads) (1)

O_2_ (ads) + e^−^ → O_2_^−^ (ads) (2)

NO_2_ (gas) + e^−^ → NO_2_^−^ (ads) (3)

NO_2_ (gas) + O_2_^−^ (ads) + 2e^−^ → NO_2_^−^ (ads) + 2O^−^ (ads) (4)

ZnO and MoSe_2_ are n-type semiconductor materials, which are characterized by electronic conductivity, and the work functions are 4.2 and 5.1 eV, respectively [23,37]. Pd is a metal material with a work function dimension of 5.12 eV [19]. Therefore, the sensing mechanism shown in Figure 8d,e can be attributed to the Pd@ZnO/MoSe_2_ Schottky barrier and n–n heterostructure interaction. The work function of Pd NPs is larger than that of ZnO and electrons will transfer from ZnO to Pd until their Fermi level is balanced. In air, electrons gather around palladium nanoparticles. When exposed to NO_2_ gas, the electrons are released, resulting in electron depletion layer thinning and sensor resistance reduction. Due to the existence of palladium nanoparticles, electron transfer is easier to achieve and improves the sensor response. This will greatly improve the sensing performance of the Pd@ZnO/MoSe_2_ nanocomposite sensor. The n–n heterojunction formed at the interface between ZnO and MoSe_2_ may be another key factor in improving the sensing performance of NO_2_ [38]. When ZnO and MoSe_2_ contact, a heterojunction is formed at their contact interfaces. Because the work function of MoSe_2_ is larger than that of ZnO, electrons will be transferred from ZnO to MoSe_2_ until the Fermi level is balanced. The electron depletion layer and the electron accumulation layer are formed at the interfaces of ZnO and MoSe_2_, respectively. When exposed to NO_2_ gas, the carrier concentration in the heterojunction further decreases, which leads to a broadening of the depletion layer at the ZnO/MoSe_2_ interface, which is beneficial to the improvement in the sensing performance of the Pd@ZnO/MoSe_2_ nanocomposite sensor.

## 5. Conclusions

The TENG was prepared by using Al-PVA and PI, and a self-powered room temperature NO_2_ sensor was developed. By converting mechanical motion into electrical energy, the TENG could be used as a power source for driving sensors. The output voltage signals of sensors exposed to NO_2_ gas were tested under 5 Hz. Experimental results showed that, compared with single ZnO and Pd@ZnO, the TENG-driven Pd@ZnO/MoSe_2_ sensor had high sensitivity and good selectivity. The enhanced sensing properties may be due to the formations of a Schottky junction and n–n heterojunction on the contact interfaces of Pd, ZnO and MoSe_2_. This work promotes the research of a self-powered gas sensor and has important significance for environmental monitoring.

## Figures and Tables

**Table 1:** Comparison of various NO_2_ sensors.

Sensor Materials	Signal Type	Concentration	Response(V_g_/V_a_)	Ref.
RGO/ZnO	Voltage	30 ppm	3.2	[29]
WO_3_	Voltage	30 ppm	2.5	[30]
In_2_O_3_/SnS_2_	Voltage	30 ppm	10.7	[31]
WO_3_/MXene	Voltage	30 ppm	4.1	[32]
Pd@ZnO/MoSe_2_	Voltage	30 ppm	11.2	This work

## Data Availability

Not applicable.
